# Evaluation of predictive and prognostic value of androgen receptor expression in breast cancer subtypes treated with neoadjuvant chemotherapy

**DOI:** 10.1007/s12672-023-00660-z

**Published:** 2023-04-26

**Authors:** Zhendong Shi, Yingxue Liu, Shichao Zhang, Shuanglong Cai, Xu Liu, Jie Meng, Jin Zhang

**Affiliations:** 1 The Third Department of Breast Cancer, Tianjin Medical University Cancer Institute and Hospital, National Clinical Research Center for Cancer, Tianjin, China; 2 Key Laboratory of Cancer Prevention and Therapy, Tianjin Medical University Cancer Institute and Hospital, Tianjin, China; 3 Tianjin’s Clinical Research Center for Cancer, Tianjin Medical University Cancer Institute and Hospital, Tianjin, China; 4 Key Laboratory of Breast Cancer Prevention and Therapy, Tianjin Medical University, Ministry of Education, Tianjin, China

**Keywords:** Breast cancer, Androgen receptor, Adjuvant chemotherapy, Pathological complete response, Disease free survival

## Abstract

**Background:**

Neoadjuvant chemotherapy is the standard treatment for local advanced breast cancer administered to shrink tumors and destroy undetected metastatic cells, thereby facilitating subsequent surgery. Previous studies have shown that AR may be used as a prognostic predictor in breast cancers, but its role in neoadjuvant therapy and the relationship with prognosis of different molecular subtypes of breast cancer need to be further explored.

**Methods:**

We retrospectively evaluated 1231 breast cancer patients with complete medical records at Tianjin Medical University Cancer Institute and Hospital who were treated with neoadjuvant chemotherapy between January 2018 to December 2021. All the patients were selected for prognostic analysis. The follow-up time ranged from 12 to 60 months. We first analyzed the AR expression in different subtypes of breast cancer and its correlation with clinicopathological features. Meanwhile, the association of AR expression and pCR of different breast cancer subtypes was investigated. Finally, the effect of AR status on the prognosis of different subtypes of breast cancer after neoadjuvant therapy was analyzed.

**Results:**

The positive rates of AR expression in HR + /HER2-, HR + /HER2 +, HR-/HER2 + and TNBC subtypes were 82.5%, 86.9%, 72.2% and 34.6%, respectively. Histological grade III (P = 0.014, OR = 1.862, 95% CI 1.137 to 2.562), ER positive expression (P = 0.002, OR = 0.381, 95% CI 0.102 to 0.754) and HER2 positive expression (P = 0.006, OR = 0.542, 95% CI 0.227 to 0.836) were independent related factors for AR positive expression. AR expression status was associated with pCR rate after neoadjuvant therapy only in subtype of TNBC. AR positive expression was independent protective factor for recurrence and metastasis in HR + /HER2- (P = 0.033, HR = 0.653, 95% CI 0.237 to 0.986) and HR + /HER2 + breast cancer (P = 0.012, HR = 0.803, 95% CI 0.167 to 0.959), but was independent risk factors for recurrence and metastasis in TNBC (P = 0.015, HR = 4.551, 95% CI 2.668 to 8.063). AR positive expression is not an independent predictor of HR-/HER2 + breast cancer.

**Conclusions:**

AR expressed the lowest in TNBC, but it could be a potential marker for the prediction of pCR in neoadjuvant therapy. AR negative patients had a higher pCR rate. AR positive expression was an independent risk factor for pCR in TNBC after neoadjuvant therapy (P = 0.017, OR = 2.758, 95% CI 1.564 to 4.013). In HR + /HER2- subtype and in HR + /HER2 + subtype, the DFS rate in AR positive patients and AR negative patients was 96.2% vs 89.0% (P = 0.001, HR = 0.330, 95% CI 0.106 to 1.034) and was 96.0% vs 85.7% (P = 0.002, HR = 0.278, 95% CI 0.082 to 0.940), respectively. However, in HR-/HER2 + and TNBC subtypes, the DFS rate in AR positive patients and AR negative patients was 89.0% vs 95.9% (P = 0.102, HR = 3.211, 95% CI 1.117 to 9.224) and 75.0% vs 93.4% (P < 0.001, HR = 3.706, 95% CI 1.681 to 8.171), respectively. In HR + /HER2- and HR + /HER2 + breast cancer, AR positive patients had a better prognosis, however in TNBC, AR-positive patients have a poor prognosis.

## Background

According to the latest global cancer burden data, there were 685, 000 female breast cancer deaths worldwide in 2020, ranking the first in female cancer incidence and mortality. Breast cancer has become the first cancer threatening the life and health of women worldwide [1]. Breast cancer is categorized into molecular subtypes by receptor expression statuses with distinctive phenotypes, including varying response to certain treatments. These subtypes are characterized by established biomarkers such as estrogen receptor (ER), progesterone receptor (PR), and human epidermal growth factor receptor 2 (HER2) [2].

At present, assessment of HR (hormone receptor, including ER and PR), as well as HER2 status, is a critical step for predictive and prognostic evaluation [3, 4]. Using the aforementioned markers, breast cancers can be classified into four subgroups: HR + /HER2–; HR + /HER2 + ; HR–/HER2 + and HR–/HER2– (triple-negative breast cancer, TNBC). Although great progress has been made in the treatment of breast cancer, it is still a major public problem that threatens the health of women. Breast cancer is a kind of highly heterogeneous disease [5]. To improve breast cancer treatment, there remains an urgent need to identify novel and alternative therapeutic targets for this disease, particularly in TNBC which systemic cytotoxic chemotherapy remains the primary pharmacological intervention.

Approximately 70–85% of breast cancers express androgen receptor (AR) [6]. AR is emerging as a new biomarker and potential therapeutic target in the treatment of breast cancer patients [7]. AR is a steroid hormone receptor, which is related to estrogen receptor, glucocorticoid receptor, progesterone receptor and mineralocorticoid receptor belong to the nuclear receptor family [8]. AR proteins are located in the cytoplasm and bind to chaperones such as heat shock in the absence of ligands, while binding to androgens can lead to conformational changes and the exposure of nuclear localization signals (NLS). AR can be activated by androgens and act as a DNA binding transcription factor, thereby regulating the expression of a variety of genes [9, 10].

The role of AR status in breast cancer is currently being widely explored. Numerous investigations have showed the inconsistent results regarding the AR expression in breast cancer. AR expression is reported to be closely associated with clinicopathological features and prognosis of breast cancer [11]. In ER-positive breast cancer, AR positivity is reported to be associated with a better prognosis [12, 13]. However, the studies reporting prognostic implications of AR expression in TNBC and HER2 positive subtypes had paradoxical results. Some studies indicated AR positivity was associated with a better outcome [14–16], others reported a worse prognosis [17–19]. Moreover, it also has been reported that there was no correlation between AR status and outcome in TNBC [20].

Neoadjuvant chemotherapy (NAC) is the standard of care for breast cancer patients with locally advanced or even with early stages. Neoadjuvant therapy not only improves breast-conserving rates in breast cancer, but is also recognized as being useful for exploring predictive biomarkers, prognostic surrogate endpoints, and treatment effects including new reagents, making it an attractive area for drug development [21].

At present, the research on the correlation between AR and neoadjuvant chemotherapy mainly focuses on TNBC. Some studies have shown that AR positive TNBC had a lower rate of pCR compared with AR negative TNBC [22, 23], However, a recent research indicated that there was no association of AR status and the pathologic responses or survival outcomes in patients with TNBC treated with neoadjuvant chemotherapy [24].Studies regarding AR as a predictor of pCR rate and survival after neoadjuvant chemotherapy according to breast cancer subtype were insufficient [25]. Therefore, further studies exploring the prognostic and predictive role of AR in patients with breast cancer subtypes are warranted. The aim of present study was to investigate AR expression in relation to clinicopathological features, molecular subtypes, pCR rate and prognosis in primary breast cancer treated with neoadjuvant chemotherapy. Understanding the complex role of AR in breast cancer subtypes would be critical in predicting the patients who would be benefit from potential targeted AR therapy.

## Materials and methods

### Patients and data collection

This retrospective study consisted of patients with breast cancer at Tianjin Medical University Cancer Institute and Hospital who were treated with neoadjuvant chemotherapy between January 2018 to December 2021. Clinicopathological data collected included: the patient's age, menstrual status, T stage, N stage, pathological type, histological grade, ER status, PR status, HER2 status, Ki-67 index, P53 status, Epidermal Growth Factor Receptor (EGFR) status, CK5/6 status, neoadjuvant therapy regimen and cycle, neoadjuvant efficacy, operation method, diagnosis time, recurrence time and metastasis time, etc.

### Inclusion criteria and exclusion criteria

#### Inclusion criteria

1. Invasive breast cancer was confirmed by biopsy pathology. 2. Neoadjuvant therapy followed by surgical treatment (modified radical mastectomy or breast conserving surgery); 3. With Complete clinical data.

#### Exclusion criteria

1.Inflammatory breast cancer or bilateral breast cancer; 2. Stage IV breast cancer; 2. Concomitant with other malignant tumors; 3. Pregnant, delivery or lactating women; 4. Received other anti-tumor therapy before neoadjuvant therapy.

### Diagnosis, immunohistochemistry technique and staging system

The 2018 ASCO-CAP guidelines were used in the evaluation of ER, PR and HER2 immunostaining. ER and PR were considered positive when ≥ 1% of cells were stained. HER2 IHC 3 + or HER2 IHC 2 + /FISH + is defined as HER2 positive, HER2 IHC 1 + or HER2 IHC 2 + /FISH- is defined as HER2 low expression, and HER2 IHC 0 is defined as HER2 negative. Ki67 was defined as high when the percentage of stained cells was ≥ 14% and low when < 14%. Regarding AR ≥ 10% and P53 ≥ 10% stained cells in nucleus were considered positive. EGFR was considered positive when ≥ 10% of cells membrane stained. Five high-power (400 ×) fields were randomly selected from the specimen, and CK5/6 positive were defined as the percentage of CK5/6 positive cells ≥ 5%. Anatomical staging was performed according to the 8th edition of the American Joint Commission Cancer (AJCC) breast cancer staging system.

### Neoadjuvant therapy and evaluation of efficacy

All the HER2 low expression and HER2 negative patients received the standard anthracycline and taxane containing regimen. The use of platinum was on the discretion of the treating physician. However, all the HER2 positive breast cancer patients received at least 4 cycles of chemotherapy combined with trastuzumab targeted therapy. Among 429 patients with HR + /HER2 + breast cancer, 155 patients received trastuzumab single target therapy and 274 patients received trastuzumab combined with pertuzumab dual-target therapy. Among 176 patients with HR-/HER2 + breast cancer, 58 patients were treated with trastuzumab single target therapy and 118 patients were treated trastuzumab combined with pertuzumab dual-target therapy. The clinical response was evaluated according to Response Evaluation Criteria in Solid Tumors (RECIST) 1.1. Patients underwent surgery after scheduled neoadjuvant therapy. Pathologic complete response (pCR) was defined as no residual invasive breast carcinoma or metastatic carcinoma in ipsilateral axillary lymph nodes. After surgery, patients were treated with standard chemotherapy, targeted therapy and endocrine therapy according to clinical guidelines.

### Follow up

Patients who completed neoadjuvant therapy in our hospital from January, 2018 to December, 2021 were followed up mainly by telephone inquiry, outpatient and inpatient medical records. The main observation was on whether the patient had local recurrence, distant metastasis and the specific time. The follow-up deadline was December 31, 2022. Disease-free survival (DFS) was defined as the interval between the date of diagnosis and the date for which relapse was confirmed or the date of the most recent clinic appointment.

### Statistical analysis

SPSS 24.0 software was used to analyze the data. Chi-square test was used to analyze the relationship between clinicopathological data and AR and pCR rate after neoadjuvant therapy. Binary Logistic Regression Analysis was used to analyze the independent clinicopathological factors related to AR and pCR rate after neoadjuvant therapy. Kaplan-Meier curve analyzed by Graphpad Prism was used to describe disease-free survival (DFS) in each subtype, and Log-Rank test was used to analyze whether AR was a risk factor for DFS. The influencing factors of DFS were analyzed by COX regression analysis. A p-value of ≤ 0.05 was considered statistically significant for all of the analyses.

## Results

### AR expression in breast cancer subtypes

A total of 1231 eligible cases of breast cancer were enrolled from January, 2018 to December, 2021, of which 917 cases were AR positive and 314 cases were AR negative, and the AR positive rate was 74.5%. There were 418 cases of HR + /HER2- breast cancer, of which 345 cases were AR positive, and the AR positive rate was 82.5%. AR was detected in 373 (86.9%) of 429 HR + /HER2 + breast cancers, and in 127 (72.2%) of 176 cases of HR-/HER2 + breast cancers. There were 208 cases of TNBC, of which 72 cases were AR positive, and the AR positive rate was 34.6% (Table 1).

**Table 1:** AR positive rate in breast cancer subtypes

Breast cancer subtype	N	N(AR +)	AR + ratio
All patients	1231	917	74.50%
HR + /HER2-	418	345	82.50%
HR + /HER2 +	429	373	86.90%
HR-/HER2 +	176	127	72.20%
TNBC	208	72	34.60%

### Correlation between AR expression and clinicopathological factors

Univariate analysis showed that AR expression was associated with older age (P = 0.022), earlier T stage (P = 0.048), lower histological grade (P = 0.034), ER positivity (P < 0.001), PR positivity (P < 0.001) and HER2 positivity (P < 0.001) in breast cancer. Binary Logistic Regression Analysis of the above related factors showed that histological grade III (P = 0.014, OR = 1.862, 95% CI 1.137 to 2.562) was an independent negative correlation factor for AR positive expression. ER positivity (P = 0.002, OR = 0.381, 95% CI 0.102 to 0.754) and HER2 positivity (P = 0.006, OR = 0.542, 95% CI 0.227 to 0.836) were independent positive correlated factors of AR expression (Table 2).

**Table 2:** Correlation between AR and clinicopathological factors

Factors	N	AR + (%)	Univariate analysis		Binary Logistic Regression Analysis	
			X^2^	P-value	OR (95% CI)	P-value
All patients	1231	917 (74.5)				
Age			5.263	0.022		
< 50	633	454 (71.7)			ref	
≥ 50	598	463 (77.4)			0.693(0.437–1.045)	0.059
Menstrual status			1.135	0.287		
Premenopausal	666	488 (73.3)				
Postmenopausal	565	429 (75.9)				
T stage			3.899	0.048		
T1, T2	943	716 (75.9)			ref	
T3, T4	288	202 (70.1)			1.096(0.578–1.652)	0.138
N stage						
N0	454	329 (72.5)	0.193	0.66		
N +	777	572 (73.6)				
Histological type			0.398	0.528		
IDC	1037	776 (74.8)				
Other	194	141 (72.7)				
Histological grade			4.517	0.034		
I, II	675	519 (76.9)			ref	
III	556	398 (71.6)			1.862(1.137–2.562)	0.014
ER status			150.939	< 0.001		
Negative	384	199 (51.8)			ref	
Positive	847	718 (84.8)			0.381(0.102–0.754)	0.002
PR status			31.275	< 0.001		
Negative	566	379 (67.0)			ref	
Positive	665	538 (80.9)			0.769(0.515–1.213)	0.077
Ki67 index			1.33	0.248		
< 14%	158	111 (70.3)				
≥ 14%	1081	806 (74.6)				
P53 status			1.675	0.196		
Negative	667	487 (73.0)				
Positive	564	430 (76.2)				
HER2 status			41.612	< 0.001		
Negative	626	417 (66.6)			ref	
Positive	605	500 (82.6)			0.542(0.227–0.836)	0.006
EGFR status			3.07	0.079		
Negative	818	622 (76.0)				
Positive	413	295 (71.4)				
CK5/6 status			0.161	0.688		
Negative	1059	791 (74.7)				
Positive	172	126 (73.3)				

*IDC* invasive ductal carcinoma

### Correlation between AR status and neoadjuvant therapy efficacy in different subtypes of breast cancer

Of all the 1231 patients, 240 cases (19.5%) achieved pCR after neoadjuvant therapy. 30 cases of 418 HR + /HER2- breast cancer patients achieved pCR, and the pCR rate was 7.2%. 106 cases of 429 HR + /HER2 + subtype achieved pCR, and the pCR rate was 24.7%. Meanwhile, the pCR rate of HR-/HER2 + subtype and TNBC subtype was 33.0% and 22.1%, respectively. In Spring et al. research [26], the pCR rate in TNBC was 32.6% (range: 20.3–62.2%). The lower pCR in this study may be due to the large tumor burden of the breast cancer patients included in this study, such as large size of the tumor, the majority patients with lymph node metastasis and with the high expression of Ki-67.

**Fig. 1:**
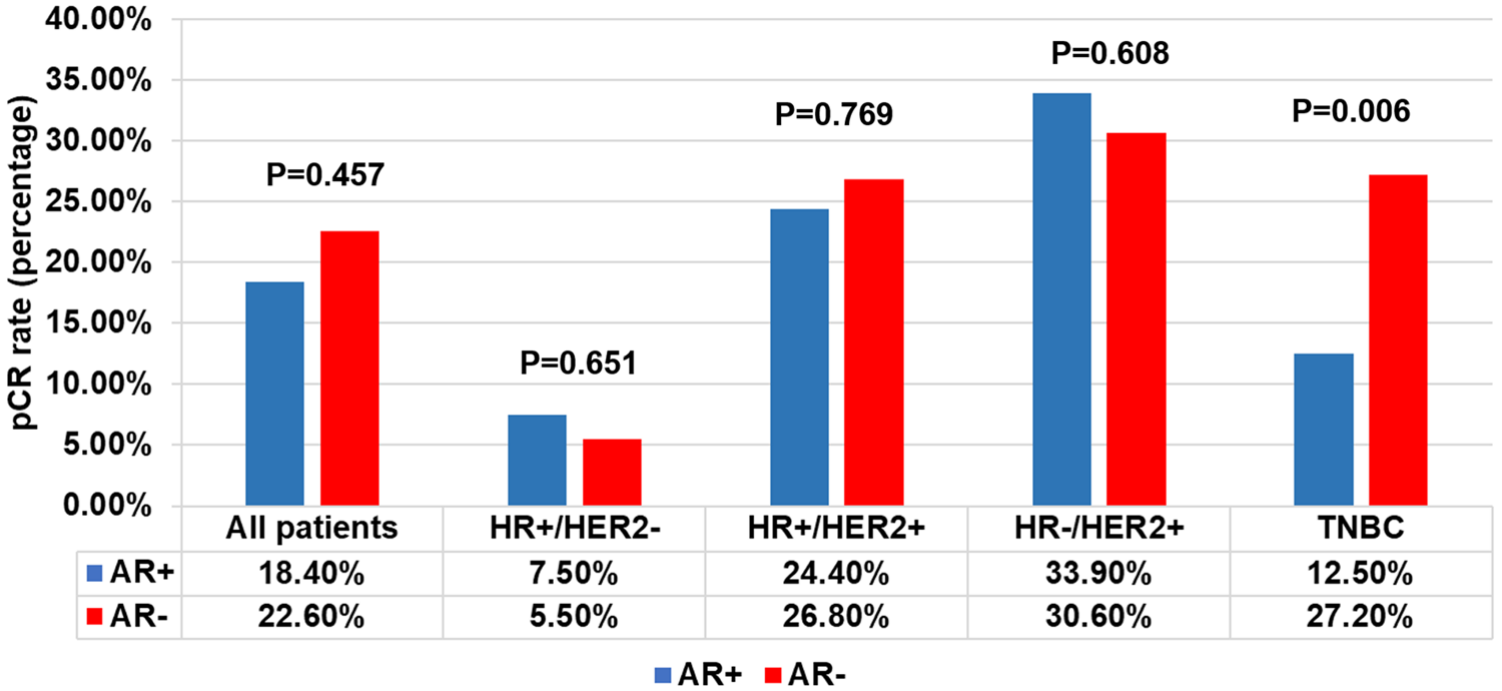
Association of AR status and pCR rate in different subtype of breast cancer

To further explore the association of AR status and pCR rate, we expanded the analysis in different breast cancer subtypes (Fig. 1). Of the 917 AR positive patients, 169 (18.4%) achieved pCR after neoadjuvant therapy. Among the AR negative patients, 71 (22.6%) achieved pCR (P = 0.457). In HR + /HER2-subtye, 26 (7.5%) of the 345 AR positive patients achieved pCR, and the pCR rate was 5.5% (4/73) in AR negative patients (P = 0.651). In HR + /HER2 + breast cancer patients, 91 (24.4%) of 373 AR positive achieved pCR, and the pCR rate was 26.8% (15/56) in AR negative patients (P = 0.769). In HR-/HER2 + subtype, 43 (33.9%) of 127 AR positive patients achieved pCR. and 15 (30.6%) of 49 AR negative patients achieved pCR (P = 0.608). Among the TNBC patients with AR positive (n = 72), 9 (12.5%) achieved pCR, and the pCR rate was 27.2% (37/136) in AR negative patients (P = 0.006).

The above results indicated that AR expression status was associated with pCR rate after neoadjuvant therapy only in TNBC. AR-negative patients had a higher pCR rate. To further investigate whether AR positive was an independent predictor for pCR rate after neoadjuvant therapy in TNBC, we performed univariate analysis and Binary Logistic Regression Analysis of pCR rate (Table 3). Univariate analysis showed that no lymph node metastasis (P = 0.013), higher histological grade (P = 0.009), HER2 IHC 0 (P = 0.013) and AR negative (P = 0.015) were more likely to achieve pCR after neoadjuvant therapy. Binary Logistic Regression Analysis of the above clinicopathological factors confirmed lymph node metastasis (P = 0.048, OR = 1.490, 95% CI 1.037 to 3.608), HER2 low expression (P = 0.034, OR = 2.713, 95% CI 1.636 to 3.798) and AR positive (P = 0.017, OR = 2.758, 95% CI 1.564 to 4.013) were independent risk factors for pCR in TNBC after neoadjuvant therapy.

**Table 3:** Univariate and Binary Logistic Regression Analysis of neoadjuvant efficacy in TNBC

Factors	N	pCR(%)	Univariate analysis		Binary Logistic Regression Analysis	
			X^2^	P-value	OR (95% CI)	P-value
All patients	208	46(22.1)				
Age			0.005	0.944		
< 50	94	21 (22.3)				
≥ 50	114	25 (21.9)				
Menstrual status			0.001	0.975		
Premenopausal	99	22 (22.2)				
Postmenopausal	109	24 (22.0)				
T stage			0.219	0.64		
T1, T2	148	34 (22.9)				
T3, T4	60	12 (20.0)				
N stage			6.227	0.013		
N0	76	24 (31.6)			ref	
N +	132	22 (16.7)			1.490(1.037–3.608)	0.048
Histological type			0.088	0.767		
IDC	166	36 (21.7)				
Others	42	10 (23.8)				
Histological grade			6.779	0.009		
I, II	112	17 (15.2)			ref	
III	96	29 (30.2)			0.732(0.507–1.465)	0.112
HER2 status			6.147	0.013		
0 expression	68	22 (32.3)			ref	
Low expression	140	24 (17.1)			2.713(1.636–3.798)	0.034
ki67 index			0.572	0.449		
< 14%	17	5 (29.4)				
≥ 14%	191	41 (21.5)				
P53 status			0.533	0.465		
Negative	82	16 (19.5)				
Positive	126	30 (23.8)				
AR status			5.911	0.015		
Negative	136	37 (27.2)			ref	
Positive	72	9 (12.5)			2.758(1.564–4.013)	0.017
EGFR status			0.004	0.949		
Negative	141	31 (22.0)				
Positive	67	15 (22.4)				
CK5/6 status			0.341	0.559		
Negative	180	41 (22.8)				
Positive	28	5 (17.8)				
NAC period			0.975	0.614		
≤ 4	16	2 (12.5)				
> 4, ≤ 6	89	21 (23.6)				
> 6	103	23 (22.3)				

*IDC* invasive ductal carcinoma, *NAC* neoadjuvant chemotherapy

### Effect of AR status on the prognosis of different breast cancer subtypes

**Fig. 2:**
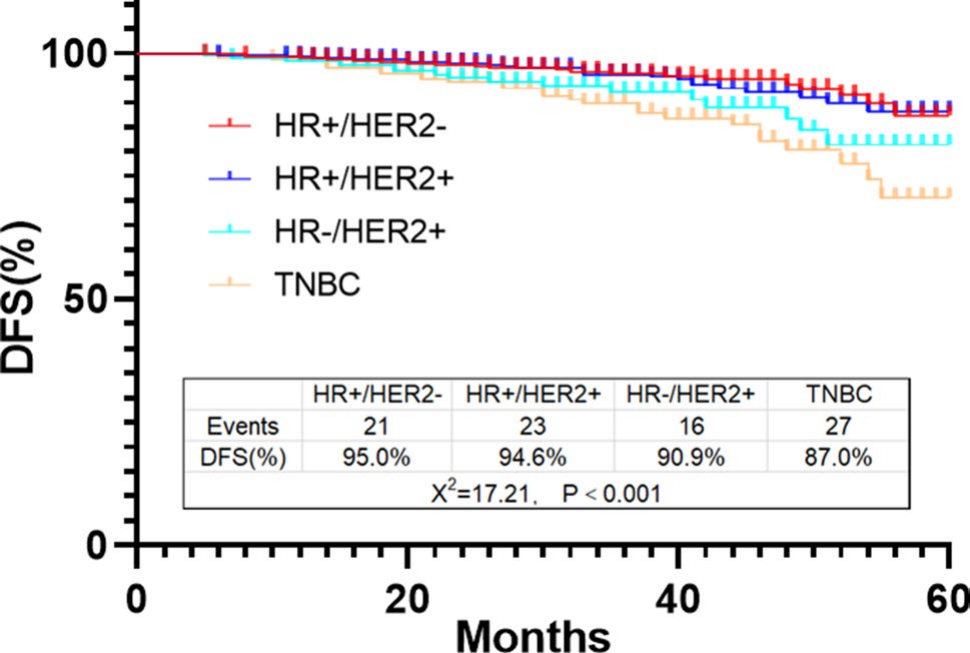
DFS rate in different subtype of breast cancer

A total of 1231 patients who completed neoadjuvant therapy from January, 2018 to December, 2021 were selected for prognostic analysis. The follow-up time ranged from 12 to 60 months, with an average of 35 months. In 418 cases of HR + /HER2- subtype, 21 cases had recurrence and metastasis, and the DFS rate was 95.0%. 23 cases had recurrence and metastasis in 429 cases of HR + /HER2 + subtype, and the DFS rate was 94.6%. In 176 cases of HR-/HER2 + subtype, 16 cases had recurrence and metastasis, and the DFS rate was 90.9%. Among 208 cases of TNBC, 27 cases had recurrence and metastasis, and the DFS rate was 87.0% (Fig. 2).

**Fig. 3:**
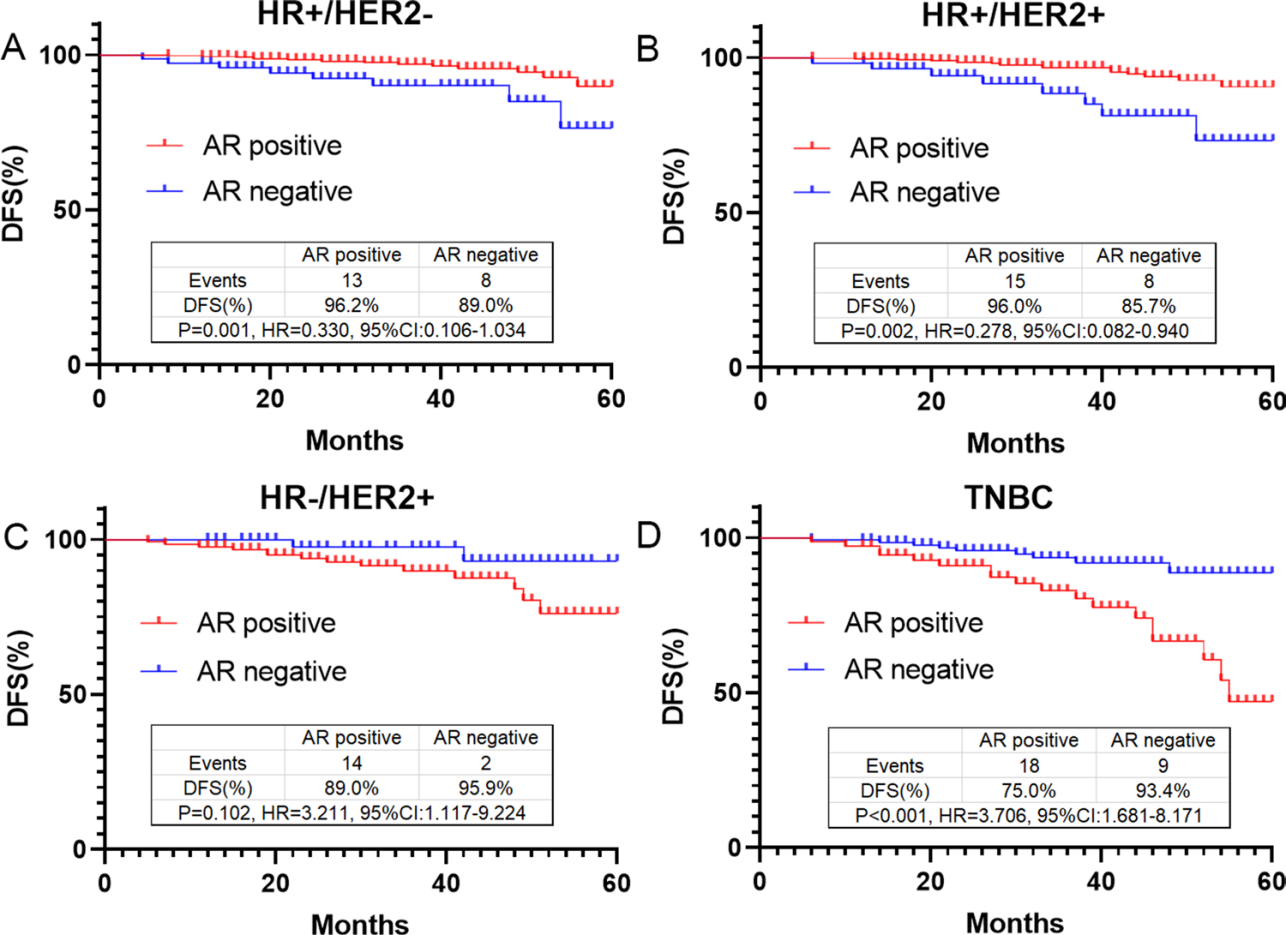
Association of 3-year DFS rate and AR status in HR + /HER2- subtype (**A**), HR + /HER2 + (**B**), HR-/HER2 + (**C**) and TNBC (**D**)

The association between AR and DFS of breast cancer subtypes was further analyzed by Log-rank test (Fig. 3). In HR + /HER2- subtype, there were 13 AR positive cases and 8 AR negative cases had recurrence and metastasis. AR positive patients had a better outcome, and the DFS rate was 96.2% vs 89.0% (P = 0.001, HR = 0.330, 95% CI 0.106 to 1.034) in AR positive cases and AR negative cases, respectively (Fig. 3A).

Similarly in HR + /HER2 + subtype, AR positive patients also had a better outcome. The DFS rate in AR positive patients and AR negative patients was 96.0% vs 85.7% (P = 0.002, HR = 0.278, 95% CI 0.082 to 0.940), respectively (Fig. 3B). However, there was no association between AR and DFS in HR-/HER2 + subtype. In TNBC subtype, AR positive patients had a worse outcome. The DFS rate in AR positive patients and AR negative patients was 89.0% vs 95.9% (P = 0.102, HR = 3.211, 95% CI 1.117 to 9.224) and 75.0% vs 93.4% (P < 0.001, HR = 3.706, 95% CI 1.681 to 8.171) respectively (Fig. 3C and D).

COX regression analysis was then used to test whether AR status was an independent predictor for the prognosis of each breast cancer subtype. Univariate analysis showed that T stage (P = 0.034), N stage (P = 0.015), histological grade (P = 0.018), p53 expression (P = 0.023) and AR status (P = 0.011) were the predictive factors of recurrence and metastasis in HR + /HER2- breast cancer. The above factors were analyzed by multivariate regression analysis. N positive (P = 0.024 HR = 3.139, 95% CI 1.425 to 7.034) and p53 positive (P = 0.038, HR = 2.675, 95% CI 1.037 to 5.983) were independent risk factors for recurrence and metastasis of HR + /HER2-breast cancer. AR positive (P = 0.033, HR = 0.653, 95% CI 0.233 to 0.986) was an independent protective factor (Table 4).

**Table 4:** Prognostic factors analysis in HR + /HER2- breast cancer

Factors	N	Events (%)	Univariate analysis		Multivariate analysis	
			X^2^	P-value	HR (95% CI)	P-value
All patients	418	21(5.0)				
Age			0.339	0.56		
< 50	213	12(5.6)				
≥ 50	205	9(4.4)				
Menstrual status			0.927	0.336		
Premenopausal	216	13(6.0)				
Postmenopausal	202	8(4.0)				
T stage			4.497	0.034		
T1, T2	319	12(3.8)			ref	
T3, T4	99	9(9.1)			2.165(0.843–4.476)	0.077
N stage			5.902	0.015		
N0	187	4(2.1)			ref	
N +	231	17(7.4)			3.139(1.425–7.034)	0.024
Histological type			0.084	0.772		
IDC	370	19(5.1)				
Others	48	2(4.2)				
Histological grade			5.564	0.018		
I、II	224	6(2.7)			ref	
III	194	15(7.7)			1.907(0.662–3.419)	0.062
ki67 index			0.066	0.797		
< 14%	47	2(4.3)				
≥ 14%	371	19(5.1)				
P53 status			5.134	0.023		
Negative	239	7(2.9)			ref	
Positive	179	14(7.8)			2.675(1.037–5.983)	0.038
HER2 status			0.218	0.641		
0 expression	159	9(5.7)				
Low expression	259	12(4.6)				
AR status			6.529	0.011		
Negative	73	8(11.0)			ref	
Positive	345	13(3.8)			0.653(0.237–0.986)	0.033
EGFR status			1.426	0.232		
Negative	288	12(4.2)				
Positive	130	9(6.9)				
CK5/6 status			1.943	0.163		
Negative	361	16(4.4)				
Positive	57	5(8.8)				
Surgery			0.005	0.943		
Conservative	82	4(4.9)				
Mastectomy	336	17(5.1)				
Radiotherapy			0.791	0.374		
Yes	348	16(4.6)				
No	70	5(7.1)				
NAC effect			0.194	0.659		
pCR	30	1(3.3)				
non-pCR	388	20(5.2)				

*IDC* invasive ductal carcinoma, *NAC* neoadjuvant chemotherapy

In HR + /HER2 + breast cancer, univariate analysis showed that T stage (P = 0.040), N stage (P = 0.049), P53 expression (P = 0.016) and AR status (P = 0.002) were the predictive factors of recurrence and metastasis. Multivariate regression analysis showed that T3, T4 (P = 0.047, HR = 1.994, 95% CI 1.148 to 3.264) and N positive (P = 0.026, HR = 2.970, 95% CI 1.364 to 5.907) was independent risk factors for recurrence and metastasis of HR + /HER2 + breast cancer, while positive AR (P = 0.012, HR = 0.803, 95% CI 0.167 to 0.959) was an independent protective factor (Table 5).

**Table 5:** Prognostic factors analysis in HR + /HER2 + breast cancer

Factors	N	Events (%)	Univariate analysis		Multivariate analysis	
			X^2^	P-value	HR (95% CI)	P-value
All patients	429	23(5.4)				
Age			0.045	0.832		
< 50	233	12(5.2)				
≥ 50	196	11(5.6)				
Menstrual status			0.086	0.769		
Premenopausal	255	13(5.1)				
Postmenopausal	174	10(5.7)				
T stage			4.211	0.04		
T1, T2	335	14(4.2)			ref	
T3, T4	94	9(9.6)			1.994(1.148–3.264)	0.047
N stage			3.873	0.049		
N0	131	3(2.3)			ref	
N +	298	20(6.7)			2.970(1.364–5.907)	0.026
Histological type			0.301	0.583		
IDC	355	20(5.6)				
Others	74	3(4.1)				
Histological grade			3.12	0.077		
I, II	244	9(3.7)				
III	185	14(7.6)				
ki67 index			2.274	0.132		
< 14%	66	1(1.5)				
≥ 14%	363	22(6.1)				
P53 status			5.576	0.016		
Negative	252	8(3.2)			ref	
Positive	177	15(8.5)			1.475(0.549–2.752)	0.139
HER2 status			0.496	0.481		
2 + /FISH +	99	9(9.1)				
3 +	116	14(12.1)				
AR status			10.11	0.002		
Negative	56	8(14.3)			ref	
Positive	373	15(4.0)			0.803(0.167–0.959)	0.012
EGFR status			0.219	0.64		
Negative	279	16(5.7)				
Positive	150	7(4.7)				
CK5/6 status			0.106	0.745		
Negative	373	20(5.4)				
Positive	56	3(5.4)				
Surgery			0.29	0.59		
Conservative	94	4(4.3)				
Mastectomy	335	19(5.7)				
Radiotherapy			0.022	0.882		
Yes	341	18(5.3)				
No	88	5(5.7)				
NAC effect			1.778	0.182		
pCR	106	3(2.8)				
non-pCR	323	20(6.2)				

*IDC* invasive ductal carcinoma, *NAC* neoadjuvant chemotherapy

In HR-/HER2 + breast cancer, N stage (P = 0.043), histological grade (P = 0.015), P53 status (P = 0.017) and neoadjuvant efficacy (P = 0.040) were determined to be the predictive factors of recurrence and metastasis by univariate analysis. Multivariate analysis indicated that N positive (P = 0.019, HR = 4.233, 95% CI 1.623 to 6.759), histological grade III (P = 0.036, HR = 2.729, 95% CI 1.206 to 6.795) and non-pCR after neoadjuvant therapy (P = 0.027, HR = 2.306, 95% CI 1.252 to 5.439) were independent risk factors for recurrence and metastasis of HR-/HER2 + breast cancer, while AR status was not a predictive factor (P = 0.151). (Table 6).

**Table 6:** Prognostic factors analysis in HR-/HER2 + breast cancer

Factors	N	Events	Univariate analysis		Multivariate analysis	
			X^2^	P-value	HR(95% CI)	P-value
All patients	176	16(9.1)				
Age			0.082	0.775		
< 50	93	9(9.7)				
≥ 50	83	7(8.4)				
Menstrual status			0.021	0.884		
Premenopausal	96	9(9.4)				
Postmenopausal	80	7(8.8)				
T stage			3.427	0.064		
T1, T2	141	10(7.1)				
T3, T4	35	6(17.1)				
N stage			4.112	0.043		
N0	60	2(3.3)			ref	
N +	116	14(12.1)			4.233(1.623–6.759)	0.019
Histological type			0.036	0.849		
IDC	146	13(8.9)				
others	30	3(10.0)				
Histological grade			5.949	0.015		
I, II	95	4(4.2)			ref	
III	81	12(14.8)			2.729(1.206–6.795)	0.036
ki67 index			0.457	0.499		
< 14%	20	1(5.0)				
≥ 14%	156	15(9.6)				
P53 status			5.708	0.017		
Negative	94	4(4.3)			ref	
Positive	82	12(14.6)			1.819(0.365–4.149)	0.102
AR status			2.062	0.151		
Negative	49	2(4.1)				
Positive	127	14(11.0)				
EGFR status			0.293	0.588		
Negative	110	9(8.2)				
Positive	66	7(10.6)				
CK5/6 status			0.662	0.416		
Negative	145	12(8.3)				
Positive	31	4(12.9)				
Surgery			0.367	0.545		
Conservative	44	3(6.8)				
Mastectomy	132	13(9.8)				
Radiotherapy			0.082	0.775		
Yes	137	12(8.8)				
No	39	4(10.3)				
NAC effect			4.203	0.04		
pCR	58	2(3.4)			ref	
non-pCR	118	14(11.9)			2.306(1.252–5.439)	0.027

*IDC* invasive ductal carcinoma, *NAC* neoadjuvant chemotherapy

In TNBC, T stage (P = 0.017), N stage (P = 0.037), histological grade (P = 0.022), AR status (P < 0.001) and neoadjuvant efficacy (P = 0.048) were proved to be predictive factors for recurrence and metastasis by univariate analysis. Multivariate analysis showed that N positive (P = 0.032, HR = 3.633, 95% CI 1.819 to 8.687) and AR positive expression (P = 0.015, HR = 4.551, 95% CI 2.668 to 8.063) and non-pCR after neoadjuvant therapy (P = 0.029, HR = 3.825, 95% CI 1.864–5.991) were independent risk factors for TNBC recurrence and metastasis (Table 7).

**Table 7:** Prognostic factors analysis in TNBC

Factors	N	Events (%)	Univariate analysis		Multivariate analysis	
			X^2^	P-value	HR (95% CI)	P-value
All patients	208	27(13.0)				
Age			0.007	0.933		
< 50	94	12(12.8)				
≥ 50	114	15(13.2)				
Menstrual status			0.004	0.949		
Premenopausal	99	13(13.1)				
Postmenopausal	109	14(12.8)				
T stage			5.632	0.017		
T1, T2	148	14(9.5)			ref	
T3, T4	60	13(21.7)			2.097(0.628–5.890)	0.066
N stage			4.345	0.037		
N0	76	5(6.6)			ref	
N +	132	22(16.7)			3.633(1.819–8.687)	0.032
Histological type			0.054	0.816		
IDC	166	22(13.3)				
Others	42	5(11.9)				
Histological grade			5.253	0.022		
I, II	112	9(8.0)			ref	
III	96	18(18.8)			1.202(0.816–2.651)	0.059
ki67 index			1.824	0.177		
< 14%	17	4(23.5)				
≥ 14%	191	23(12.0)				
P53 status			0.328	0.567		
Negative	82	12(14.6)				
Positive	126	15(11.9)				
HER2 status			3.807	0.051		
0 expression	98	8(8.2)				
Low expression	110	19(17.3)				
AR status			14.083	< 0.001		
Negative	136	9(6.6)			ref	
Positive	72	18(25.0)			4.551(2.668–8.063)	0.015
EGFR status			2.126	0.145		
Negative	141	15(10.6)				
Positive	67	12(17.9)				
CK5/6 status			0.049	0.825		
Negative	180	23(12.8)				
Positive	28	4(14.3)				
Surgery			0.002	0.964		
Conservative	47	6(12.8)				
Mastectomy	161	21(13.0)				
Radiotherapy			0.162	0.687		
Yes	153	19(12.4)				
No	55	8(14.5)				
NAC effect			3.897	0.048		
pCR	46	2(4.3)			ref	
non-pCR	162	25(15.4)			3.825(1.864–5.991)	0.029

*IDC* invasive ductal carcinoma, *NAC* neoadjuvant chemotherapy

## Discussion

The role of AR signaling in breast cancer has received much attention. Our study found that AR was widely highly expressed in HR + breast cancers, and the expression rates in HR + /HR- and HR + /HR- were 82.5% and 85.9%, respectively, while the expression rate in TNBC was only 34.6%. Multivariate correlation analysis further showed that histological grade I and II, ER positive and HER2 positivity were independent positively correlated factors for AR expression. Previous studies indicated AR was mainly expressed in ER-positive, PR positive and non-TNBC [15, 27]. However, the reason of AR low expression in TNBC is unknown. Perhaps future research will reveal this issue.

Neoadjuvant therapy has become an important treatment mode for locally advanced breast cancer. After neoadjuvant therapy, the patients can achieve tumor downstaging, improve the breast-conserving rate, and effectively observe the therapeutic drug sensitivity. pCR after neoadjuvant therapy was significantly associated with the improvement of OS (overall survival) and DFS, and it was the most objective evaluation index of the efficacy of neoadjuvant therapy [28].

Different subtypes of breast cancer had great differences in response to neoadjuvant therapy. In present study, HR-/HER2 + breast cancer has the highest pCR rate (33.0%), while HR + /HER2- breast cancer had the lowest pCR rate (6.7%). Some patients with HER2 + breast cancer in this study were treated with trastuzumab alone because pertuzumab has only been accessible in China since 2019. To date, there were few studies on the predictive function of AR in neoadjuvant response of breast cancer, especially in HER2-positive breast cancer and HR-positive breast cancer. By analyzing the correlation between AR status and pCR after neoadjuvant therapy in different subtypes of breast cancer, we found that only the TNBC pCR rate was correlated with AR expression status (P = 0.006), and AR-negative patients had a higher pCR rate. In a previous study of 55 patients with HER2-positive breast cancer who received trastuzumab plus pertuzumab neoadjuvant therapy, pCR positively related to high expression levels of AR (OR 33.145, 95% CI 2.803 to 391.900, P = 0.005) [29]. However, there was no correlation between AR expression and pCR rate in HR + /HER2-, HR + /HER2 + or HR-/HER2 + breast cancer (P = 0.651, P = 0.769 and P = 0.608, respectively) in this study.

To further investigate whether AR expression was an independent predictor of pCR rate after neoadjuvant therapy in TNBC, logistic multivariate analysis was performed, and both AR negative and HER2 IHC 0 were independent protective factors for high pCR rate in TNBC. Despite its clinical aggressiveness, TNBC was generally considered to be more sensitive to chemotherapy compared to other histological subtypes.

With the development of new HER2-targeted therapeutic drugs in recent years, more and more research focus has turned to the low expression of HER2. Although patients with low expression of HER2 are diagnosed as HER2 negative, their tumor cells also have different degrees of HER2 protein expression on the surface, which may affect the prognosis of patients. However, new antibody-drug conjugates (ADC), such as DS-8201 (Trastuzumab Deruxtecan), can kill HER2-low tumor cells through their unique drug mechanism to produce therapeutic effect [30]. In this study, in TNBC patients with HER2 low expression, the pCR rate and DFS rate are lower than those with HER2 IHC 0 expression. Perhaps new ADC drugs can provide treatment options for such patients. In addition, although HER2 status was important for the breast cancer treatment, other ErbB receptors (including EGFR, HER3 and HER4) were also considered to play a crucial role in breast cancer pathogenesis. The co-expression profile of ErbB receptors might also be useful in predicting prognosis of AR-positive breast cancer patients. Therefore, HER3 and HER4 might represent attractive new markers for the application of novel targeting strategies to improve breast cancer treatment efficacy [31].

To investigate the impact of AR expression on the outcome of breast cancer treated with neoadjuvant chemotherapy, we analyzed the correlation between AR status and DFS in molecular subtypes of neoadjuvant breast cancer. The results showed that AR-positive patients had a good prognosis in HR + /HER2 − and HR + /HER2 + breast cancers. Further COX univariate and multivariate analysis showed that AR positive expression was an independent protective factor for recurrence and metastasis of the above two breast cancer subtypes.

Previous studies showed that most luminal breast cancers expressed AR, and this expression suggested a good prognosis. A study to determine the clinical significance of AR expression in luminal breast cancer showed that AR-positive cases had better results in terms of time to recurrence (TTR) and disease specific survival (DSS) [12]. Another independent study showed that high AR expression in HR + tumors was associated with reduced lymphocyte infiltration, a marker of better prognosis [32]. A large-scale clinical and gene expression meta-analysis by Bozovic-Spasojevic et al. confirmed that AR positivity improved DFS and OS in HR + breast cancer patients [15].

HR-/HER2 + breast cancer accounts for about 15–20% of all breast cancers, and our study showed that AR positive rate was 72.2% in this type of breast cancer, and the log-rank test showed that AR-positive patients had poor prognosis, which was partially consistent with some previous studies [33, 34]. COX multivariate regression analysis showed that AR was not a predictor of recurrence and metastasis of HR-/HER2 + breast cancer. This may be due to the sample size, tumor heterogeneity and treatment differences included in the survival analysis.

The DFS analysis of TNBC showed that AR positive expression indicated poor prognosis. Furthermore, COX regression analysis confirmed that AR positive expression was an independent risk factor for TNBC recurrence and metastasis (P = 0.015). In this study, AR-positive TNBC was found to have a low pCR rate and poor DFS after neoadjuvant chemotherapy. Certain controversies remain regarding AR in TNBC survival prediction. In a meta-analysis, AR expression in TNBC was associated with longer DFS and OS [35]. In a study based on 116 metastatic TNBC cases, AR positive expression was found to be an independent prognostic protective factor, as AR positivity (AR > 10%) was associated with higher 5-year survival [14]. However, in a study of 263 patients with primary early TNBC, AR expression and its correlation with prognosis was evaluated. AR expression was associated with worse outcomes and an increased risk of late distal DFS events in TNBC [36]. More interestingly, an international multicenter study evaluated AR status in tumor tissues of 1407 TNBC patients from six different countries. The results indicated that AR status appeared a population-specific pattern associated with OS. AR-positive was a marker of better prognosis in the US and Nigerian cohorts, but a marker of worse prognosis in the Norwegian, Irish, and Indian cohorts, and neutral in the UK cohort [37]. We proposed that population-dependent differences in the biological effects of AR depend on differences in potential modifiers, such as AR splice variants, epigenetic factors, or tumor microenvironment, which may affect patient prognosis and response to AR-targeted agents. Although the role of AR as a prognostic predictive biomarker in TNBC was controversial, increasing evidence suggests that AR-positive TNBC may be responsive to therapeutic agents targeting AR, thus bringing a new dawn to the treatment of TNBC.

In conclusion, this study systematically investigated the association of AR expression with pCR after neoadjuvant therapy and DFS in different subtypes of breast cancer. AR expression was highest in HR + /HER2 + breast cancer and lowest in TNBC. Histological grade III, ER positivity and HER2 positivity were independent factors associated with AR positivity. There was a correlation between AR expression and pCR rate only in TNBC. AR expression was associated with the outcome of HR + /HER2- and HR + /HER2 + breast cancer and TNBC. AR-positive patients had a good prognosis after neoadjuvant therapy in HR-positive breast cancer, but a poor prognosis in HR-negative breast cancer. AR positive expression was an independent protective factor for the outcome of HR + /HER2-, HR + /HER2 + breast cancer, and an independent risk factor for TNBC. However, there are still many limitations. First, this was a retrospective single-center study, and there was no specific treatment for each subtype of breast cancer. Some HER2-positive patients received single trastuzumab targeted therapy. Second, the follow-up time was too short to assess long-term survival, no significant difference in OS data was obtained. We will continue to follow up for the patients’ survival. If possible, a multi-center randomized study will be conducted for data analysis in future.

## Data Availability

The datasets used and analyzed during the current study are available from the corresponding author on reasonable request.

## Abbreviations

AR: Androgen receptor
ER: Estrogen receptor
PR: Progesterone receptor
HR: Hormone receptor
HER2: Human epidermal growth factor receptor 2
TNBC: Triple negative breast cancer
NLS: Nuclear localization signals
RECIST: Response evaluation criteria in solid tumors
pCR: Pathologic complete response
NAC: Neoadjuvant chemotherapy
DFS: Disease free survival
OS: Overall survival
TRR: Time to recurrence
DSS: Disease specific survival
ADC: Antibody–drug conjugates
